# CRBP-1 over-expression is associated with poor prognosis in tongue squamous cell carcinoma

**DOI:** 10.1186/s12885-018-4249-1

**Published:** 2018-05-02

**Authors:** Yue Chen, Tian Tian, Min-Jie Mao, Wei-Ye Deng, Hao Li

**Affiliations:** 1 Sun Yat-sen University Cancer Center, State Key Laboratory of Oncology in South China, Collaborative Innovation Center for Cancer Medicine, 651 Dong Feng Road East, Guangzhou, Guangdong 510060 China; 2 Department of head and neck surgery, Sun Yat-sen University Cancer Center, 651 Dong Feng Road East, Guangzhou, Guangdong 510060 China; 3 Department of Clinical laboratory, Sun Yat-sen University Cancer Center, 651 Dong Feng Road East, Guangzhou, Guangdong 510060 China; 4 Department of Radiation Oncology, The University of Texas MD Anderson Cancer Center, 1515 Holcombe Blvd., Houston, Texas 77030 USA

**Keywords:** CRBP-1, Tongue squamous cell carcinoma, Expression, Prognosis, Knockdown, Proliferation, Invasion

## Abstract

**Background:**

Tongue squamous cell carcinoma (TSCC) is one of the most common malignancies of oral squamous cell carcinomas. Cellular retinol binding protein-1 (CRBP-1) as a carrier protein transports retinol from the liver storage site to peripheral tissue. Up-regulated expression of CRBP-1 is associated with some tumor types such as prostate cancer, breast cancer and ovarian cancer as reported, but its role in TSCC remains uncertain.

**Methods:**

In this study, an integrated bioinformatics analysis based on the multiple cancer microarray data sets available from Oncomine database was conducted to view the differential expression of CRBP-1 between TSCC and the adjacent non-tumorous tissues. Quantitative real-time polymerase chain reaction (qRT-PCR), western blotting (WB) and immunohistochemical (IHC) assays were performed to investigate CRBP-1 expression in 101 paraffin-embeded TSCC tissues and 48 pairs of freshly frozen tissues. Kaplan-Meier curve and univariate and multivariate Cox-regression analysis were used to estimate the association between CRBP-1 expression and patients’ prognosis. Then western blotting, MTT, transwell migration and invasion assays were performed in TSCC cell lines to investigate the effects of CRBP-1 on cellular proliferation and invasion.

**Results:**

Compared with the matched adjacent non-tumorous tissues, the expression of CRBP-1 was significantly up-regulated in TSCC tissues, which correlated with the differentiation state (*P* = 0.003), N classification (*P* = 0.048), the clinical stage (*P* = 0.048) and death (*P* = 0.001). The Kaplan-Meier curve showed that TSCC patients with higher CRBP-1 expression levels had lower overall survival rates than those with lower CRBP-1 expression levels. A univariate and multivariate analysis demonstrated that CRBP-1 was an independent prognostic factor (*P* < 0.05). Furthermore, we knocked down CRBP-1 expression and observed that TSCC cell proliferation and invasion in vitro were significantly blocked, as determined by MTT and transwell assays.

**Conclusions:**

Up-regulated expression of CRBP-1 is associated with poor prognosis in TSCC, so it might potentially serve as an additional prognostic marker, and the inhibition of CRBP-1 might provide new therapeutic approaches for TSCC.

## Background

Tongue squamous cell carcinoma (TSCC) is one of the most common types of oral squamous cell carcinomas (OSCC). TSCC is highly invasive and characterized by early metastasis and poor prognosis [1–4]. Although therapeutic targets of TSCC have been improved, the mortality rates of TSCC patients are still high [5–7].

Regional lymph node metastasis has been known as an adverse prognostic factor of TSCC [2, 8, 9]. Poorly differentiated cancer is more likely to have cervical metastasis, positive margins after resecting and poor survival [10]. Histologic risk is another important prognostic predictor in OSCC [11]. A better comprehension of the connection between certain molecular alterations and histological differentiation may inject fresh blood into studying the mechanisms of tumor occurrence and development, and provide an effective strategy for the diagnosis, treatment and prevention of TSCC.

Retinol (known as Vitamin A) and its metabolic products are necessary for many biochemical reactions and affect epithelial cell proliferation and differentiation [12]. The biological effects of retinol are primarily mediated by all trans-retinoic acid receptors (RARs) and 9-cis retinoic acid receptors (RXRs) [13, 14]. Currently, researchers have focused much attention on the roles of retinoic acid binding proteins and cellular retinol binding proteins (CRABPs and CRBPs) in carcinogenesis. Retinoid intracellular transportation and retinoid-induced cellular activities in physiology are under regulation of CRABPs and CRBPs. Among them,CRBP-1 takes an important part in the uptake and subsequent esterification of retinol and improving bioavailability and transcriptional activities. In addition, CRBP-1 plays a vital role in the embryonic development, growth, vision and survival of vertebrates [15, 16].

Recent studies found that CRBP-1 was down-regulated in certain human cancer tissues, including prostate cancer [17], breast cancer [18], endometrial cancer [19] and ovarian cancer [20], and that CRBP-1 was up-regulated in lung adenocarcinoma [15] and laryngeal cancer [21]. However, few studies focused on the clinicopathological features of CRBP-1 expression levels in human TSCC. Therefore, the goal of our study was to determine whether CRBP-1 influenced tumor progression and overall survival (OS) rates in TSCC patients. To find out the relationship between CRBP-1 and TSCC, qRT-PCR, western blotting and immunohistochemistry were conducted to examine CRBP-1 expression in a batch of human TSCC tissues and the matched non-tumorous tissues. We also carry out some cell experiments to investigate the effects of CRBP-1 on tumor cells’ proliferation and migration.

## Methods

### Patients and tumor tissue samples

An immunohistochemistry analysis was conducted on tongue tumor specimens collected from 101 patients who were diagnosed with tongue squamous cell carcinoma and had surgical resection between September of 2005 and February of 2009 at the Head and Neck Department of the Sun Yat-sen University Cancer Center. All patients obtained a biopsy before surgery, and the diagnosis of TSCC was confirmed by the Pathology Department of Sun Yat-sen University Cancer Center according to the World Health Organization (WHO) histological criteria. Patients bearing other primary tumors at the same time were excluded in this study. All patients’ clinicopathological records, based on the clinical and pathologic staging system of the American Joint Committee on Cancer (AJCC), were reviewed. Overall survival (OS), defined as the time from the surgery to the date of death or the last follow-up, were used to measure the patients’ prognosis. Detailed information of the 101 patients is shown in Table 1.

**Table 1:** Clinical characteristic and CRBP-1 express of 101 patient samples of TSCC

Characteristics	Number of cases (%)
Age (years)	
≤45	27(26.7)
> 45	74(73.2)
Gender	
Male	61(60.4)
Female	40(39.6)
Tumor size (cm)	
≤2	73(72.3)
> 2	28(27.7)
Differentiation status	
Well	72(71.3)
Moderate Poor	24(23.8)
Poor	5(4.9)
T classification classification	
T1	73(72.3)
T2	27(26.7)
T3	1(1.0)
T4	0
N classification	
N0	78(77.2)
N1	10(9.9)
N2	13(12.9)
N3	0
M classification	
M0	100(99.0)
M1	1(1.0)
Clinical stage	
I	63(62.4)
II	15(14.8)
III	10(9.9)
IV	13(12.9)
Relapse	
Yes	23(22.8)
No	78(77.2)
Death	
Yes	27(26.7)
No	74(73.3)
Expression of CRBP-1	
Low expression	49(48.5)
High expression	52(51.5)

48 pairs of fresh TSCC tissues and matching adjacent non-tumorous tongue tissues (located more than 1 cm away from the tumor) collected from TSCC patients after resection at the Sun Yat-sen University Cancer Center from 2011 to 2015 were used for qRT-PCR and western blotting analysis.

### Database

The Oncomine microarray database (http://www.oncomine.org) was used to screen CRBP-1 expression in the paired TSCC samples. “CRBP-1” was used as a keyword in the Oncomine search, “Cancer vs. Normal Analysis” was used as the primary filter, and “Tongue squamous cell carcinoma” was chosen as the cancer type. The up-regulated mRNA levels of CRBP-1 were shown in multiple data sets including Talbot’s, Estilo’s and Ye’s datasets. We also screened the gene expression levels in paired head and neck squamous carcinoma (HNSCC) samples (normal vs. carcinoma tissues). The up-regulated mRNA levels of CRBP-1 were shown in multiple datasets including Cromer’s, Peng’s, Toruner’s and Ginos’ data sets. The CRBP-1 expression data were log-transformed, median-centered per array, and the standard deviation (SD) was normalized to one per array.

### Cell culture and generation of stably transfected cell lines

The human TSCC cell line CAL-27 was maintained in Dulbecco’s Modified Eagle’s Medium (DMEM) supplemented with 10% fatal bovine serum (FBS). The human TSCC cell lines (SCC-9, SCC-15 and SCC-25) were cultured in a mixture of DMEM (Invitrogen) and Ham’s F12 medium and DMEM supplemented 25 mM Hepes, 100 μg/mL penicillin/streptomycin, 4 mM L-glutamine and 10% FBS (Gibco). Tca8113 was grown in RPMI-1640 (Invitrogen) supplemented with 10% FBS, 15 mM Hepes, 100 μg /mL penicillin and 2.5 μg /mL. The lentiviruses containing CRBP-1 shRNA were bought from GenePharma (Shanghai, China). The transfection was conducted and stable cell lines (CAL-27 or SCC-25 cells) expressing CRBP-1 shRNAs were selected for 2 weeks with 3 μg/ml puromycin. The sequences targeting CRBP-1 were 5′- GTGGATTGAGGGTGATGAA-3′(#1) and 5’-GTCTGTCTCATTGCCTTGT-3′(#2).

### Immunohistochemistry (IHC)

IHC was performed to study CRBP-1 expression in TSCC tissues with the same method described above. At first, paraffin-embedded tissue sections were deparaffinized and rehydrated in a series of descending ethanol concentrations. Then, the sections were treated with 3% hydrogen peroxide for 15 min at room temperature and placed in a high-pressure cooker for antigen retrieval. The slides were incubated with rabbit anti-CRBP-1 (1:100; Abcam, U.S.A.) in a humidified chamber at 4 °C overnight, then incubated with an anti-rabbit secondary antibody at 37 °C for 30 min, following by treatment with diaminobenzidine tetrahydrochloride (DAB) and counterstaining with hematoxylin. An isotypic antibody dilution was used for each sample as the negative control under the same experimental conditions.

The CRBP-1 immunostaining results were scored by two independent pathologists based on both percentage and intensity of positively stained tumor cells under double-blind conditions. The percentage of positive cells was scored as follows:“0”(0–5%), “1”(5–25%), “2”(25–50%), “3”(50–75%) and “4”(75–100%). The intensity were scored as follows: “0”(no staining), “1”(weakly staining), “2”(moderately staining) and “3”(strongly staining). The final score was calculated by multiplying the staining intensity score by the positive percentage score. We devided the patients into high or low expression group with the median score. The final score of low expression group was < 8 and the final score of high expression group was ≥8. [22]

### Quantitative real-time polymerase chain reaction (qRT-PCR)

Total RNA was extracted from fresh tissues and TSCC cells using a TRIZOL reagent according to the manufacturer’s instructions (Thermo Fischer Scientific, Waltham, MA, U.S.A.). 2 μg of the extracted RNA pretreated with RNase-free DNase were used for the cDNA synthesis primed with random hexamers. The obtained cDNAs were applied to a real-time PCR analysis to evaluate the relative expression levels of CRBP-1 and β-actin (used as an internal control) with the following primers: 5′- CTACAATGAGCTGCGTGTGG -3′ (forward); 5’-AGGTACTCCTCGAAATTCTCGTT-3′ (reverse) for CRBP-1; 5′- CATGTACGTTGCTATCCAGGC -3′(forward); and 5′- CTCCTTAATGTCACGCACGAT -3′(reverse) for β-actin. The quantitative PCR was performed using a Light Cycler real-time quantitative PCR system 480 (Roche, Germany).

### Western blotting assay

Both fresh tissues and TSCC cells were homogenized in a RIPA lysis buffer, and the lysates were cleared by centrifugation. The obtained proteins were then transferred to a polyvinylidene difluoride membrane that was incubated with a CRBP-1 primary antibody (1:1000, Abcam, U.S.A.) or β-actin (1:1000, Abcam, U.S.A.) and with 5% skim milk. The membranes were incubated with anti-rabbit IgG or anti-mouse IgG and then visualized by the ECL system (Santa Cruz Biotechnology, Dallas, TX, U.S.A.).

### MTT assay

CAL-27 and SCC-25 cells were implanted in 96-well plates with 1500 cells per well after transfection. Cellular proliferation was measured by means of MTT assay at 1, 2, 3, 4, 5 and 6 days, and their absorbance values were measured at 490 nm wavelength with a spectrophotometric plate reader.

### Transwell assay

CAL-27 and SCC-25 cells were respectively trypsinized and counted, and then implanted in the top chamber with the matrigel-coated membrane (24-well insert; pore size, 8 μm; BD Biosciences) with 2 × 105 cells per well in medium without serum. Medium supplemented with serum was used as a chemical attractant in the lower chamber. After incubated for 24 h, the cells that had migrated or invaded through the membrane to the lower surface were fixed, stained, and counted under the inverted microscope. Cells were counted for normalization of matrigel invasion activity by counting the siRNA-treated cells by microscopy 24 h after plating the equal number of the cells on culture plates.

### Statistical analysis

Data were presented as mean ± SD. The Chi-square test,Student’s t-test and Fisher’s exact test were used for comparisons between groups. The Kaplan-Meier test and cox regression models were used to analyze the survival rates of the TSCC patients. All data were analyzed with SPSS software (version 21; SPSS Inc., Chicago, IL, U.S.A.). A two-sided *p* value < 0.05 was regarded as statistically significant.

## Results

### CRBP-1 expression levels in tongue tissues and the adjacent non-tumorous tissues

**Fig. 1:**
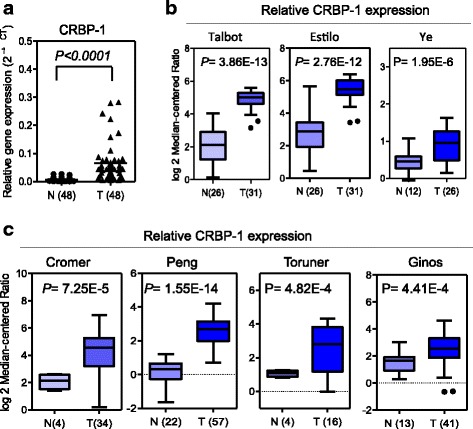
Expression of CRBP-1 mRNA in human TSCC tissues and matched adjacent non-tumorous tissues. **a** The relative CRBP-1 mRNA expression levels significantly increased in TSCC tissues (T) compared with those of the matched adjacent non-tumorous tissues (ANT)(*n* = 48, *P* < 0.001). The mRNA expression level of CRBP-1 was measured by qRT-PCR. **b** CRBP-1 is overexpressed in human TSCC tissues(T) than the adjacent normal tissues(N) in multiple cancer microarray data sets available from Oncomine. **c** CRBP-1 is overexpressed in HNSCC tissues(T) than the adjacent normal tissues(N) in multiple cancer microarray data sets available from Oncomine. Data is presented as mean ± S.D. **P < 0.01 versus the corresponding control groups

**Fig. 2:**
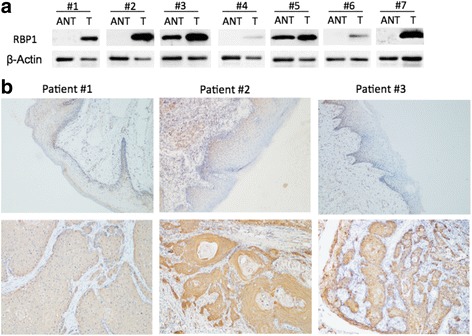
Expression of CRBP-1 protein in human TSCC tissues and matched adjacent non-tumorous tissues. **a** The relative CRBP-1 protein expression in 7 pairs of TSCC tissues (T) and the matched adjacent non-tumorous tissues (ANT). GAPDH was measured as the loading control. **b** Immunohistochemical staining of CRBP-1 in three pairs of representative TSCC tissues (T) and the adjacent non-tumorous tissues (ANT)(× 100)

To explore the expression levels of CRBP-1 in TSCC tissues (T) and in the adjacent non-tumorous tissues (ANT), the qRT-PCR assay was used to detect the levels of mRNA in 48 paired tongue samples, and were compared to the matched adjacent non-tumorous tissues. As shown in Fig. 1a, the CRBP-1 mRNA expression levels were significantly higher in 48 pairs of the TSCC tissues compared to those of the matched ANT tissues. Next, we analyzed the expression of CRBP-1 in TSCC and HNSCC in the multiple cancer microarray data sets available from Oncomine (http://www.oncomine.org), and found that the mRNA expressions of CRBP-1 in TSCC and HNSCC tissues were markedly higher than those in the matched normal non-tumorous tissues, and some normal tissues had very low CRBP-1 expression with a negative value (Fig. 1b and c). We observed that the 7 pairs of human TSCC tissues had significantly higher expression levels than found in their matched control, which was consistent with Western blot data (Fig. 2a). To further investigate the CRBP-1 expression levels in TSCC, we performed an immunohistochemical analysis of 101 paraffin-embeded TSCC tissues, which involved 89 cancers with the matched ANT tissues. Our results manifested that 95.5%(85/89) of the tumor tissues expressed significantly higher CRBP-1 levels than those of the matched ANT tissues. Three pairs of representative slides are displayed in Fig. 2b.

### Correlation between CRBP-1 expression and clinicopathological features

Next, we analyzed the potential correlation between the CRBP-1 protein expression levels of 101 TSCC surgical specimens and the clinicopathological features of the TSCC patients. In Table 2, we observed that 51.5% (52/101) of the cases exhibited high CRBP-1 expression, while 48.5%(49/101) of the cases showed relatively low CRBP-1 expression. The samples consisted of 62.4%(63/101) of the cases in clinical stage I, 14.8%(15/101) of the cases in clinical stage II, 9.9%(10/101) of the cases in clinical stage III and 12.9%(13/101) of the cases in clinical stage IV.

**Table 2:** Correlation between CRBP-1 expression and clinicopathological variables of 101TSCCcases

	CRBP-1 expression		
	Low	High	*P*
Age (years)			0.685
≤45	14	13	
> 45	35	39	
Gender			0.327
Male	32	29	
Female	17	23	
Tumor size			0.795
≤2 cm	36	37	
> 2 cm	13	15	
Differentiation status			0.003
Well	40	32	
Moderate	5	19	
Poor	4	1	
T classification			0.795
T1	36	48	
T2+T3+T4	13	15	
N classification			0.048
N0	42	36	
N1+N2+N3	7	16	
M classification			1.000
M0	49	51	
M1	0	1	
Clinical stage			0.048
I-II	36	48	
III-IV	13	15	
Relapse			0.134
Yes	8	15	
No	41	37	
Death			0.006
Yes	7	20	
No	42	32	

**Fig. 3:**
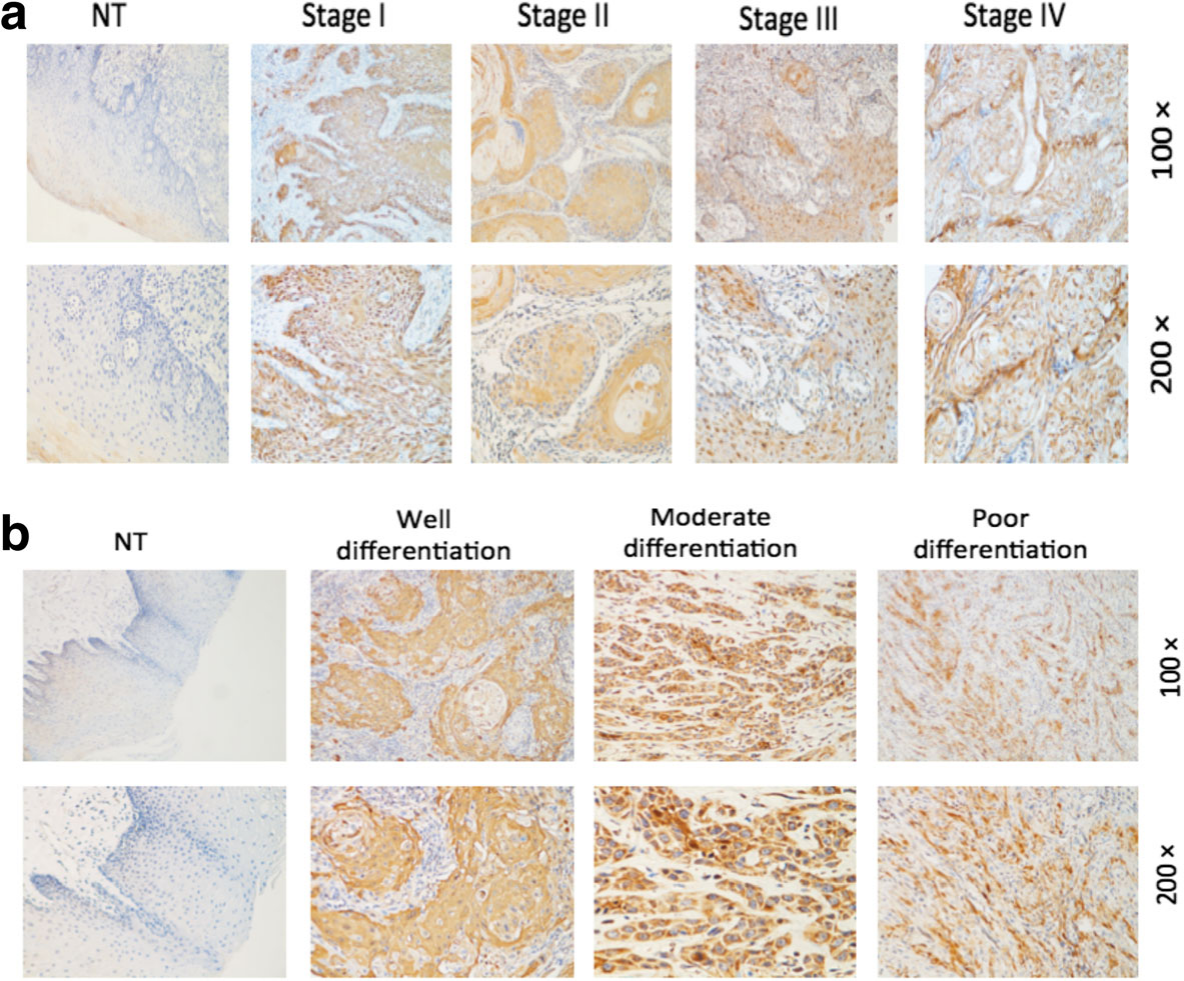
Correlation between CRBP-1 expression in TSCC and clinicopathological features. **a** Increased expression of CRBP-1 in advanced stages of TSCC. Representative IHC analysis of CRBP-1 expression in TSCC specimens (T) and the matched adjacent non-tumorous tissues (ANT) of different clinical stages. **b** CRBP-1 expression in TSCC tissues with different differentiation states

The relationship between the clinical and pathological characteristics of TSCC patients and the levels of CRBP-1 expression are described in Table 2. The results showed that the expression levels of CRBP-1 significantly correlated with multiple variables, including the differentiation state (*P* = 0.003), N classification (*P* = 0.048), clinical stage (P = 0.048) and death (*P* = 0.006), but not with age (*P* = 0.685), gender (*P* = 0.327), tumor size (*P* = 0.795), T classification (P = 0.795) and M classification (*P* = 1.000). The representative IHC-stained slides displaying the adverse connections between the tumor clinical states and CRBP-1 expression levels are shown in Fig. 3a Likewise, high expression levels of CRBP-1 were observed in the TSCC tissues with poor differentiation in Fig. 3b.

### CRBP-1 expression and patient survival analysis

**Fig. 4:**
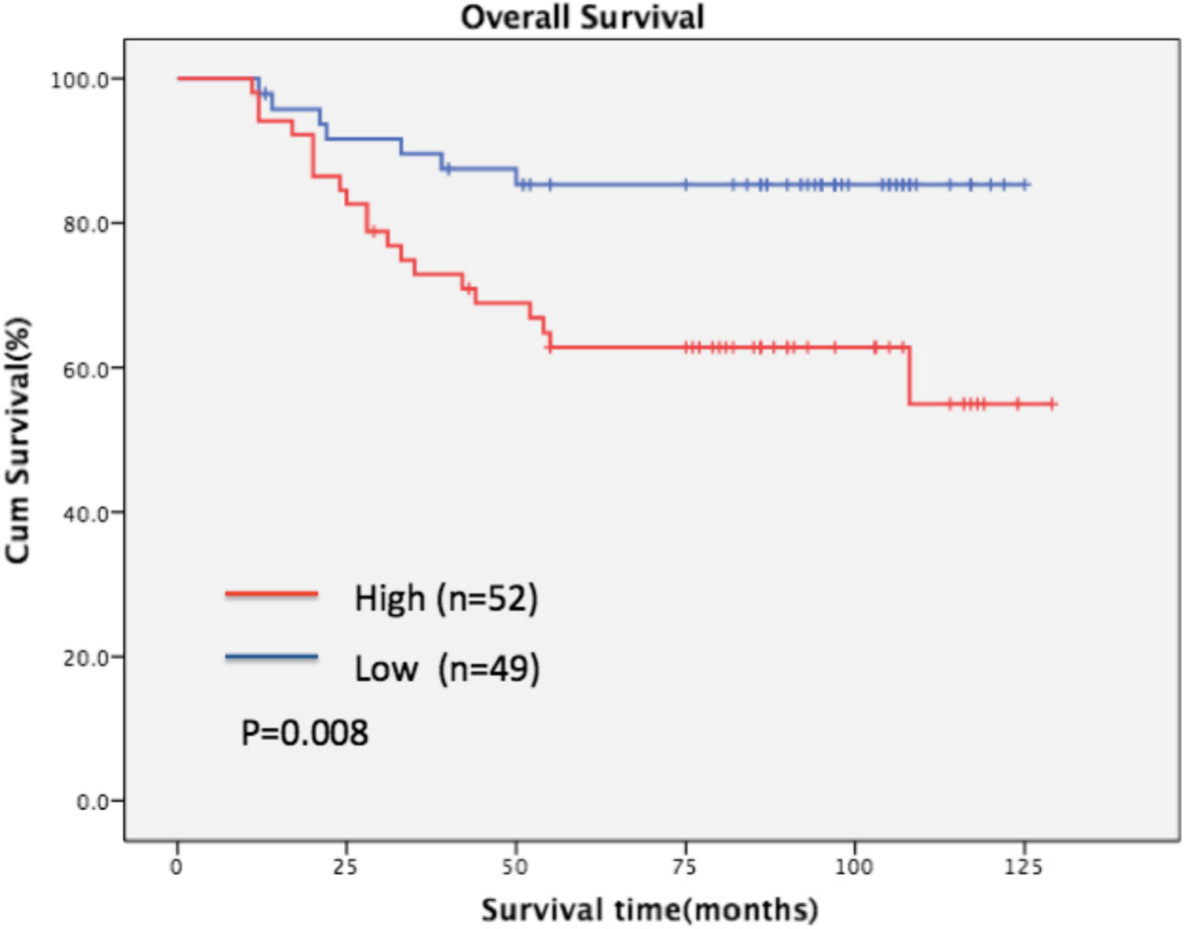
Kaplan-Meier survival curves for TSCC patients with high CRBP-1 expression (red line) versus low CRBP-1 expression (blue line). The overall survival of patients with high or low CRBP-1 expression

Patient survival analysis revealed that the overall survival (OS) in TSCC patients was different among 101 patients in accordance with CRBP-1 expression levels. High expression of CRBP-1 was correlated with a shorter overall survival time (*P* = 0.008) as shown in Fig. 4.

To investigate the independent prognostic value of CRBP-1 expression levels, a univariate and multivariate Cox regression analysis were performed (Table 3). Our results found that CRBP-1 expression, tumor size, differentiation state and clinical state were especially associated with the overall survival rates of TSCC patients. Further, CRBP-1 expression levels, the differentiation states and the clinical states were responsible for the poor OS in TSCC patients as determined by a multivariate Cox regression analysis.

**Table 3:** Univariable and multivariable analysis of overall survival of TSCC patients

Variables	Univariable analysis			Multivariable analysis		
	HR	95%CI	P	HR	95%CI	P
CRBP-1 expression (low vs. high)	3.011	1.272–7.126	0.012	2.753	1.140–6.650	0.024
Age (≤45 vs. > 45)	1.693	0.641–4.477	0.288			
Gender (male vs. female)	0.664	0.312–1.413	0.288			
Tumor size (≤2 cm vs. > 2 cm)	2.788	1.309–5.937	0.008	1.653	0.716–3.818	0.239
Differentiation stage	2.627	1.523–4.531	0.001	3.357	1.726–6.529	< 0.001
Clinical stage (I + II VS. III + IV)	4.317	2.010–9.276	< 0.001	34.328	1.977–9.477	< 0.001

### CRBP-1 expression in TSCC cell lines and CRBP-1 shRNA

**Fig. 5:**
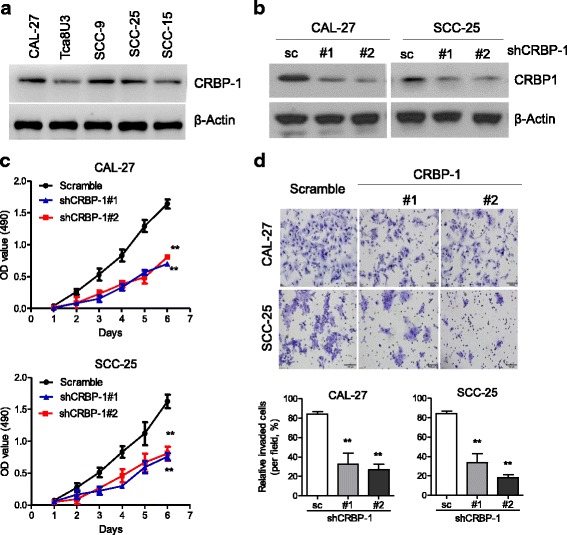
Knockdown of CBRP-1 reduced the cell proliferation and invasion ability in CAL-27 and SCC-25 cells. **a** The relative expression levels of CRBP-1 in TSCC cell lines were evaluated by western blotting. **b** Immunoblotting analysis of CRBP-1 in CAL-27 and SCC-25 cells transduced with two specific shRNA. **c** Growth curves of CAL-27 and SCC-25 cells with knockdown of CRBP-1. Scramble (sc): the lentiviral vector with a scrambled sequence that does not target any mRNA. **d** Images (upper panel) and quantification (lower panel) of invaded TSCC cells treated with melatonin for 24 h were analyzed in a transwell matrix penetration assay. Scar bar: 100 μm. Data is presented as mean ± S.D. **P < 0.01 versus the corresponding control groups

We then investigated CRBP-1 expression in CAL-27, Tca8113, SCC-25, SCC-9 and SCC-15 cell lines using western blotting analysis. Among the five TSCC cell lines analyzed, CAL-27 and SCC-25 cells had obviously high expression of CRBP-1 at protein levels (Fig. 5a). To achieve significant experimental results, CRBP-1 was stably knocked down in CAL-27 and SCC-25 cells which exhibited lower expression levels of CRBP-1 using lentiviral CRBP-1 shRNA cells compared to their control cells (Fig. 5b). Thus, the shCRBP-1 was successful in reducing CRBP-1 protein levels in these cell lines.

### Functional consequences of reduction CRBP-1 expression in vitro

Because cancer is usually characterized by uncontrolled cell proliferation, we first examined the proliferation feature of the control cells and CRBP-1-knockdown cells in CAL-27 and SCC-25 cell lines respectively. MTT assay revealed that the proliferation of CRBP-1-knockdown cells was significantly inhibited (Fig. 5c). It is known that regional lymph node metastasis is an adverse prognostic factor of TSCC. The ability of cancer cells to invade from their primary site and then into tissues is a crucial step in the formation of metastasis, so we then tested the invasive capacity of CRBP-1-knockdown cells using a transwell invasion assay. As a pro-metastasis role for CRBP-1, the number of cells invading through the membranes was significantly reduced in CRBP-1-knockdown cells in comparison to the control cells (Fig.d).

## Discussion

CRBP-1, a member of cellular retinol-binding proteins(CRBPs),belongs to the family of fatty acid-binding proteins, which widely expresses in many tissues [23]. Ghyselinck et al. found that CRBP-1 is essential in liver vitamin A esterification and storage in their study of mice [24]. Aberrant CRBP-1 expression has been found closely connected with different tumors and affected patient’s prognosis. Some researches discovered that CRBP-1 was downregulated in hepatocellular carcinoma [25], endometrial carcinoma [19], ovarian carcinoma [20] and breast carcinoma [18]. On the contrary, CRBP-1 was upregulated in astrocytic gliomas [26], leiomyosarcoma [27] and lung adenocarcinoma [15]. Moreover, decreased expression of CRBP-1 was found relevant to shorter survival time in endometrial carcinoma, ovarian carcinoma and breast carcinoma, while increased expression of CRBP-1 was verified to damage survival time in astrocytic gliomas [26], leiomyosarcoma [27] and lung adenocarcinoma [15]. Some reserchers also found that aberrant CRBP-1 expression related to the differentiation status of breast carcinoma [18], endometrial carcinoma [19] and larynx carcinoma [28]. Although CRBP-1 has been found overexpressed in Talbot’s, Estilo’s and Ye’s datasets in TSCC and Cromer’s, Peng’s, Toruner’s and Ginos’ data sets in HNSCC through microarray studies, but no one of them deepened their researches to dig out the relationship between CRBP-1 expression and the prognosis of TSCC patients and the role of CRBP-1 in TSCC cell lines.

In our study, CRBP-1 expression was investigated at transcription and translation levels in matched TSCC specimens by qPT-PCR and western blotting analysis. The mRNA and protein levels of CRBP-1 increased in tumorous tissues compared to those in the matched non-tumorous tissues. Because of individual difference, the expression of CRBP-1 in TSCC tissues and the matched adjacent normal tissues was different. Some tumor tissues with low expression were almost equal to the normal tissues with high expression. But CRBP-1 was significantly overexpressed in TSCC tissues than the adjacent normal tissues compared by the Student’s t-test (*p* < 0.05). Moreover, the CRBP-1 expression levels in the immunohistochemical analysis increased in 95.5% of the TSCC tissues (85/89), compared with those in the matched adjacent non-tumorous tissues. All of these experimental results confirmed that CRBP-1 was overexpression in TSCC tissues and it was in accordance with the search results from Oncomine.

In the Kaplan-Meier survival analysis, the CRBP-1 expression levels strongly correlated with TSCC patient survival rates. Patients with higher expression levels of CRBP-1 had a markedly decreased survival rate. Consistently, Elena et al. found the same results in lung adenocarcinoma [15]. Furthermore, we discovered that CRBP-1 expression, together with the differentiation state and clinical state, was an independent risk factor for TSCC patients by the Cox hazard ratio regression analysis.

We also found that CRBP-1 expression is closely connected with the differentiation status, T classification,clinical stage and death. So up-regulation of CRBP-1 expression might play an important role in tumor differentiation and lymph node metastasis. Under normal conditions, CRBP-1 not only regulates intracellular retinol trafficking and bioconversion, but also facilitates its biological functions [12]. Specifically, retinol can stimulate the proliferation and differentiation of epithelial cell. High expression levels of CRBP-1 in TSCC were correlated with increased CRBP- 1 gene copy numbers and were also parallel to increased tumor grades, suggesting that high expression levels of CRBP-1 reflects a more aggressive and dedifferentiated phenotype of squamous cell carcinoma. These results are consistent with Elena et al.’s study. They found that 62.3% of adenocarcinomas expressed high level of CRBP-1 which associated with increased tumor grade and reduced OS [15].

We wondered whether CRBP-1 made a great contribution to tumor proliferation and metastasis. To find out the answer, MTT and transwell assay were conducted in CAL-27 and SCC-25 cell lines with CRBP-1 knocked down. The result revealed that the proliferation and invasion of CRBP-1-knockdown cells was significantly inhibited. A recent study got the similar result in which they found significant inhibition of proliferation of A549 cells transfected with CRBP-1comparing to the control group.

We ascertained that CRBP-1 was distinctly upregulated in TSCC tissues and CRBP-1 upregulation was closely contected with reduced survival of TSCC patients. Besides, CRBP-1 expression of TSCC patients was in relation to tumor differentiation and lymph node metastasis. In addition, in vitro studies revealed that knocking down CRBP-1 inhibit the proliferation and invasion of CAL-27 and SCC-25 cell lines. However, further research is necessary to help us clarify the molecular mechanism of CRBP-1 in TSCC. To summarize, our results declare that CRBP-1 can serve as a prognostic biomarker of TSCC and a potential therapeutic target for TSCC treatment.

## Conclusion

In this study, we found that CRBP-1 expression levels increased in patients with TSCC, and high expression levels of CRBP-1 indicated a poor prognosis. Knockdown of CRBP-1 weaken the proliferation and invasion ability of TSCC cells. Our results suggest that CRBP-1 may be a promising prognostic biomarker of TSCC patients and therapeutic target for TSCC treatment.

## Abbreviations

AJCC: American Joint Committee on Cancer
CRBP-1: Cellular retinol binding protein-1
HNSCC: Head and neck squamous carcinoma
OS: Overall survival
OSCC: Oral squamous cell carcinoma
qRT-PCR: Quantitative reverse transcription polymerase chain reaction
SD: Standard deviation
TSCC: Tongue squamous cell carcinoma
WHO: World Health Organization
